# Metabolic rates, swimming capabilities, thermal niche and stress response of the lumpfish, *Cyclopterus lumpus*

**DOI:** 10.1242/bio.036079

**Published:** 2018-08-16

**Authors:** Malthe Hvas, Ole Folkedal, Albert Imsland, Frode Oppedal

**Affiliations:** 1 Research Group of Animal Welfare, Institute of Marine Research, 5984 Matredal, Norway; 2 Department of Biology, University of Bergen, 5007 Bergen, Norway; 3 Akvaplan-niva, Iceland Office, Akralind 4, 201 Kopavogur, Iceland

**Keywords:** U_crit_, Respirometry, Aerobic scope, Temperature, Size effects, Behaviour, Cortisol

## Abstract

The lumpfish (*Cyclopterus lumpus*) is a semi-pelagic globiform teleost native to the North Atlantic with a ventral suction disc that allows for attachment onto surfaces. Some local populations are in decline and the species has recently become important in salmonid sea cages as cleaner fish. Little is known about the basal physiology of the lumpfish, and a characterization of thermal performance, aerobic capacity, swimming behaviour and stress response is therefore warranted. In the present study, swim tunnel respirometry was performed on lumpfish acclimated to 3, 9 or 15°C. Higher temperatures were also attempted, but at 18°C their behaviour became erratic and 15% of the fish died over 3 weeks of acclimation. Water current tolerance was assessed in two size classes (∼75 g and ∼300 g) both with and without the ability to voluntarily use the ventral suction disc. Lastly, blood samples were taken from resting, exhausted and recovered fish to assess haematological effects of exercise stress. Lumpfish had relatively low aerobic scopes that increased slightly with temperature. Critical swimming speed was poor, increasing within the tested temperatures from 1.3 to 1.7 body lengths s^−1^ in 300 g fish. They struggled to remain sucked onto surfaces at currents above 70–110 cm s^−1^, depending on size. Acute stress effects were modest or non-existent in terms of changes in cortisol, lactate, glucose, erythrocytes and ion balance. These results describe a typical sluggish and benthic species, which is contradictory to the pelagic nature of lumpfish in large parts of its lifecycle.

## Abstract (precis)

**Summary:** Assessment of aerobic capacity and swimming behaviour of the lumpfish with swim tunnel respirometry at different acclimation temperatures and fish sizes, and measurements of haematological parameters in relation to exercise stress.

## INTRODUCTION

The lumpfish (*Cyclopterus lumpus*) is a globiform teleost native to the North Atlantic, where it is found in both pelagic waters and coastal regions (Blacker, 1983; Daborn and Gregory, 1983). It is an opportunistic forager of large planktonic organisms in the surface and mid waters, and occasionally also feeds on benthic invertebrates in the proximity of seaweeds (Davenport, 1985).

The pelagic nature of the lumpfish during the majority of its life cycle may seem paradoxal. It lacks a swim bladder as seen in other related cottoid teleosts that are primarily benthic, and pelagic fish normally either possess swim bladders or are powerful swimmers. However, the morphology of the lumpfish is indicative of a fish built for manoeuvrability and precision rather than speed and athleticism (Davenport and Kjørsvik, 1986). Furthermore, the lumpfish possesses a ventral suction disc that allows it to adhere onto surfaces such as rocks and seaweed, which may be an important strategy for energy saving, and a security measure against rough current conditions in shallow coastal areas, but is of no use in open waters.

Lumpfish are targeted by fisheries for their roe and also get taken as bycatch from other fisheries, which has resulted in notable population declines in recent years in the north-eastern Atlantic and the Gulf of St. Lawrence (Lorance et al., 2015; Gauthier et al., 2017). Furthermore, a rapidly growing trend is to use lumpfish in Atlantic salmon sea cages as cleaner fish, since they will feed on salmon lice (*Lepeophtheirus salmonis*) (Imsland et al., 2014; Powell et al., 2017). However, anecdotal reports of very high unaccounted mortalities in marine sea cages have led to ethical concerns about their utilization as cleaner fish. Farm locations are chosen based on environmental conditions that allow Atlantic salmon to thrive, and it is currently underappreciated that lumpfish may not necessarily cope in similar environments owing to fundamental physiological differences between the two species.

The basal physiology of lumpfish is poorly studied and is currently limited to acute hypoxia and thermal tolerance (Ern et al., 2016), and ontogenetic effects on aerobic capacity (Killen et al., 2006). Hence, more knowledge of their physiological response in relation to environmental change and stress should be useful in management and conservation efforts of wild populations (e.g. Carey, 2005; Cooke et al., 2012, 2013; McKenzie et al., 2016). In addition, a better understanding of the environmental limits of lumpfish is crucial for development of legislation for their ethical use in aquaculture.

A widespread framework to assess physiological performance in specific environmental conditions is the aerobic scope (AS), which is defined as the difference between resting and maximum rate of aerobic metabolism (Fry, 1971). The AS provides a quantification of the available aerobic energy to perform fitness related activities such as foraging, growth, gonad development and locomotion (Fry, 1971; Priede, 1985; Claireaux and Lefrancois, 2007). Hence, conditions that limit AS should, in theory, be suboptimal.

Another useful experimental assessment of fish performance is to test swimming capabilities in swim tunnels where the critical swimming speed (U_crit_) is obtained by incrementally increasing water current velocity until fatigue is reached (Brett, 1965; Plaut, 2001). In the case of lumpfish, such observations may shed some light on the semi-pelagic nature of this species.

In the present study, swim tunnel respirometry was performed on lumpfish acclimated to 3, 9 or 15°C. Swim tunnel respirometry allows for the measurement of U_crit_, as well as both resting and maximum rates of oxygen uptake as proxies for aerobic metabolism, and hence also for a subsequent calculation of the AS. Since metabolism and locomotory capabilities of poikilothermic fish are greatly dependent on temperature, the derived AS and U_crit_ may therefore reveal the ideal thermal conditions for lumpfish (e.g. Fry, 1971; Clark et al., 2013; Lefevre, 2016).

In addition to a typical U_crit_ protocol, lumpfish were also tested for their ability to utilize the suction cup to attach onto surfaces in progressively stronger water currents, while behavioural preferences for swimming versus attachment were assessed. Finally, to further elicit physiological adaptations in response to the environment, the effects of exhaustive exercise stress and subsequent recovery were investigated by analyses of haematological parameters. This should give some insight into the stress response, the osmotic consequences in relation to activity, and the anaerobic capacity of lumpfish.

Since the lumpfish has been regarded as a cold water species (Ern et al., 2016), it was hypothesized that high temperatures would compromise AS and U_crit_. Overall U_crit_ was expected to be low owing to the globiform morphology combined with the suction ability that may be used as an alternative to swimming. In addition, a modest stress response was expected since the lumpfish appears unathletic and hence has limited benefits from a strong mobilization of the respiratory system in potential dire situations.

## RESULTS

### Temperature effects

**Fig. 1.**
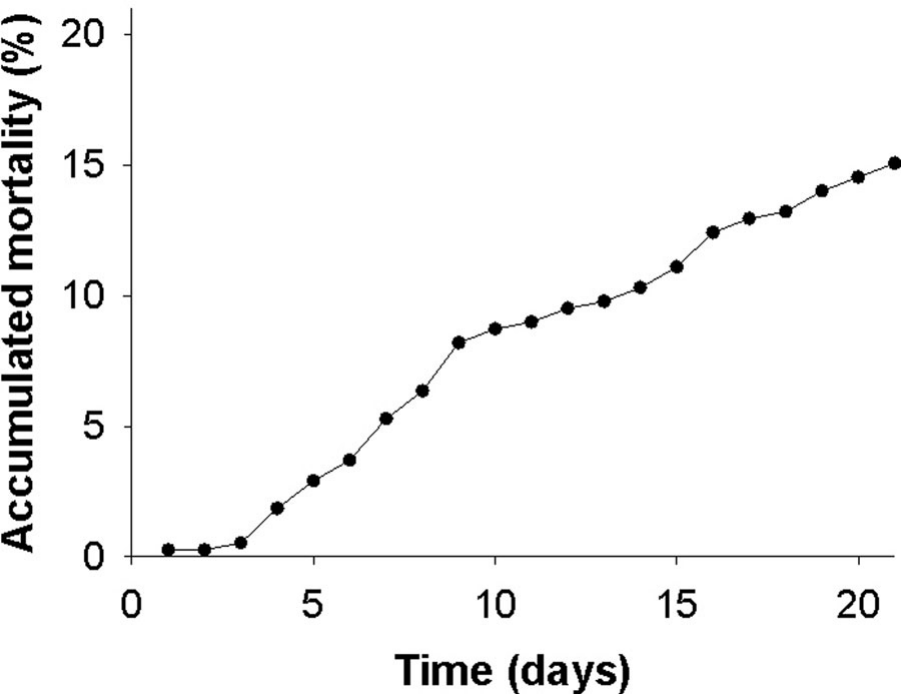
**The accumulated mortality of lumpfish over 3 weeks of exposure to 18°C in the holding tanks.**

No mortalities occurred when keeping lumpfish at 3, 9 and 15°C. However, at 18°C acclimation, the accumulated mortality in the holding tank reached 15% over 3 weeks (Fig. 1). Although the lumpfish were still observed eating at 18°C, their behaviour was more erratic, leading to less fish attached to surfaces and the majority swimming at higher than usual speeds. In addition, some individuals swam upwards at the surface with their heads protruding into the air. Chaotic group swimming at this temperature caused fish collisions, which resulted in wounds on the head and back in some individuals. The surviving fish at 18°C were returned to 9°C for an additional 3 weeks. In this period, three individuals died, which corresponded to an accumulated mortality of 0.8%. On the first day after returning to a colder temperature, behaviour returned to normal, with a large proportion of the lumpfish attaching to surfaces and the remaining swimming at a slower pace.

It should be noted that cataract formation was observed in several fish kept at 15 and 18°C, however, the extent of this occurrence was not systematically quantified in this study.

**Fig. 2.**
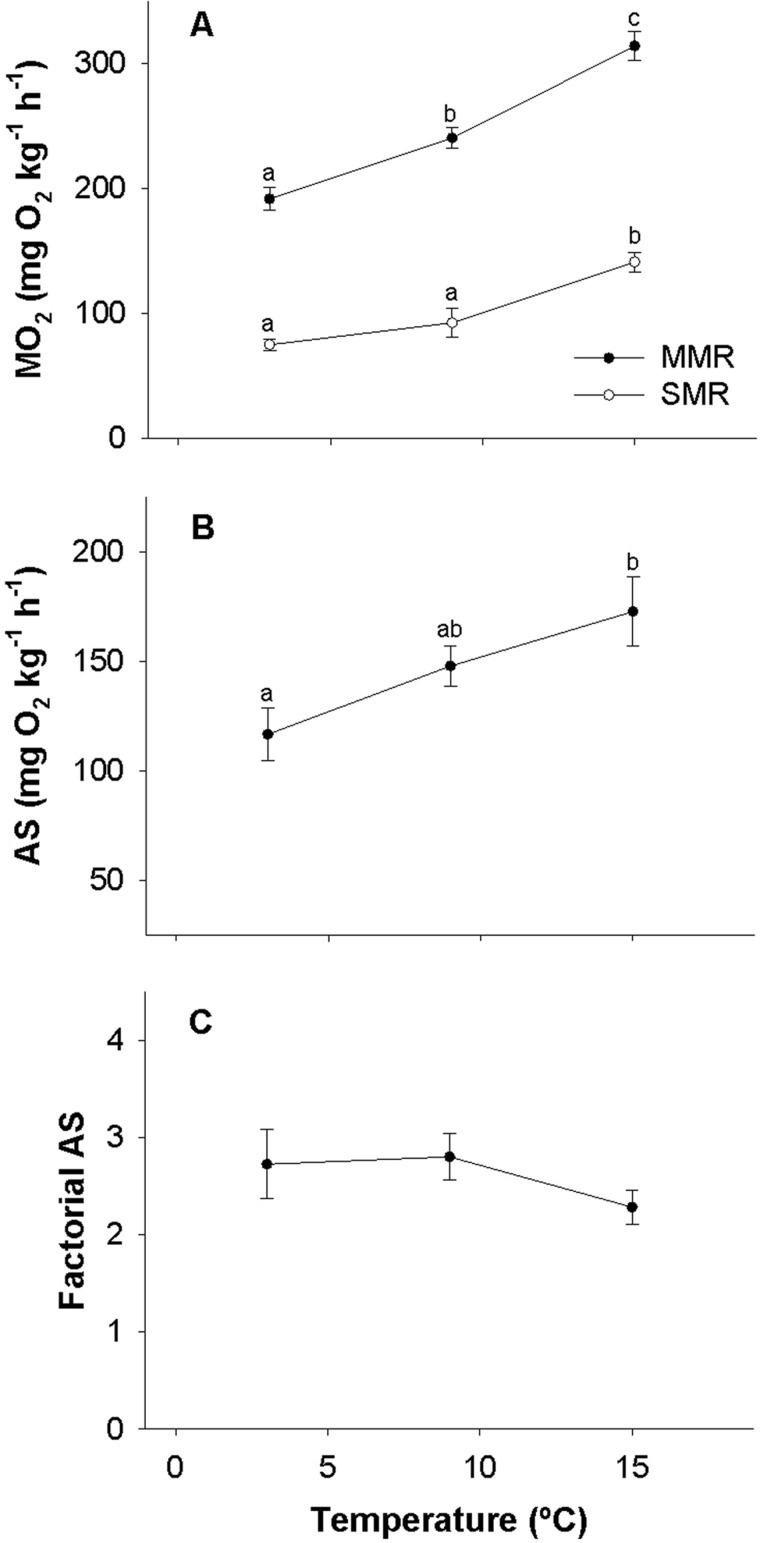
**Metabolic rates of lumpfish.** (A-C) The SMR and MMR (A), AS (B) and factorial AS (C) in lumpfish acclimated to different ecologically relevant temperatures. Statistical differences are indicated with different letters. *N*=8. Data are mean±s.e.m.

The standard metabolic rate (SMR) of lumpfish increased with acclimation temperature with a temperature coefficient (Q_10_) of 1.7 between 3 and 15°C, and a larger Q_10_ of 2.0 between 9 and 15°C (Fig. 2A). The maximum metabolic rate (MMR) also increased with temperature, but with a similar Q_10_ of ∼1.5 between the same test temperatures (Fig. 2A). The resulting AS was 117±12, 148±9 and 173±16 mg O_2_ kg^−1^ h^−1^ at 3, 9 and 15°C, respectively, where a significant difference was found between 3 and 15°C (Fig. 2B). The factorial AS was statistically similar between acclimation temperatures with a pooled average of 2.6 (Fig. 2C).

**Fig. 3.**
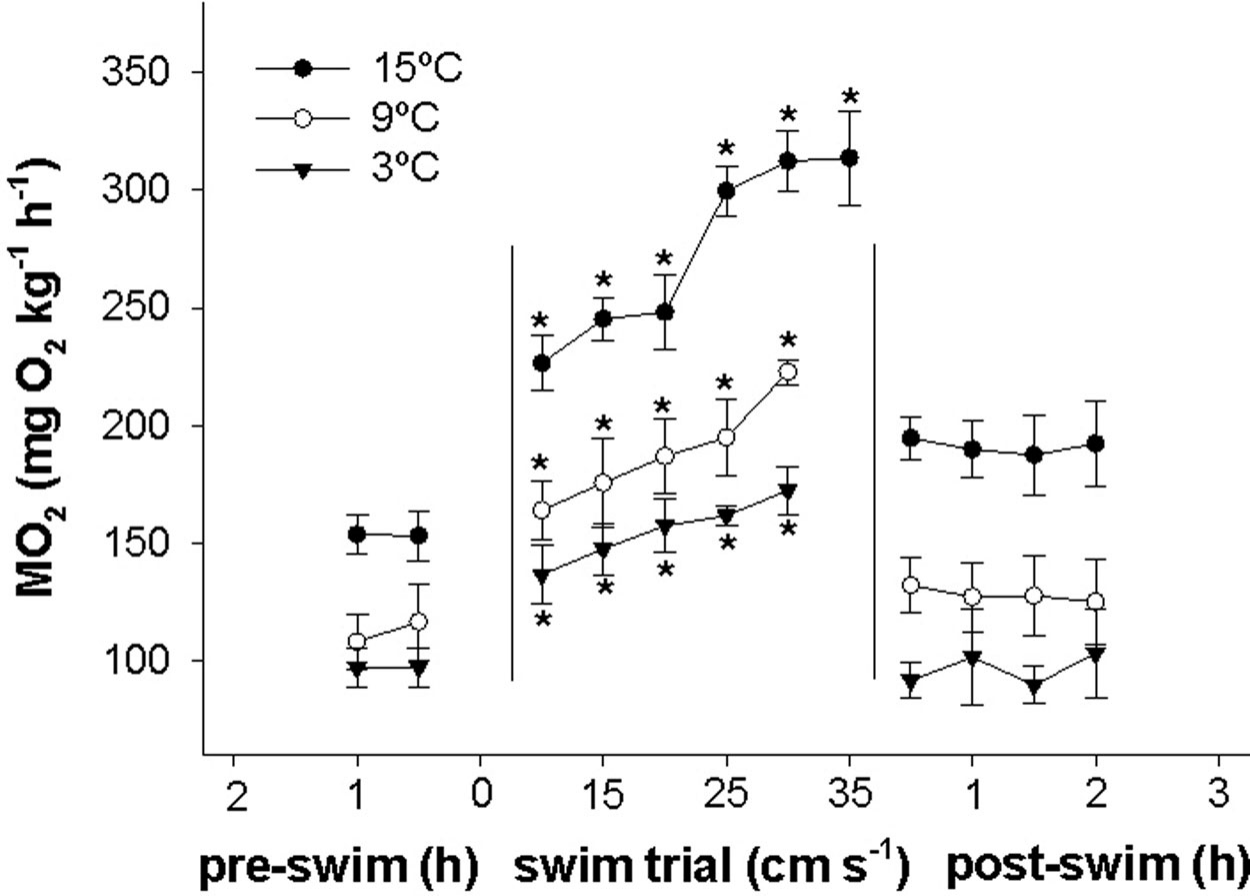
**MO_2_ of lumpfish before, during and after swim trials at 3, 9 and 15°C.** The first two points at each temperature correspond to the routine MO_2_ before swimming was initiated. The following points are MO_2_ while swimming at increasingly higher speeds until maximum capacity. The final four points at each temperature represents MO_2_ over a 2 h period after swimming to exhaustion. Asterisks indicate a significant difference in MO_2_ compared to 1 h before swim trials at each respective temperature. *N*=8. Data are mean±s.e.m.

The oxygen uptake rate (MO_2_) prior, during and after swim trials at the three test temperatures are depicted in Fig. 3. A distinct difference between temperatures can be seen with a higher MO_2_ at higher temperatures at various activity levels. No excess post-exercise oxygen consumption (EPOC) was observed following swim trials at 3, 9 and 15°C, where MO_2_ immediately returned to a statistically similar level as measured 1 h pre-exercise and remained stable for the following 2 h.

**Fig. 4.**
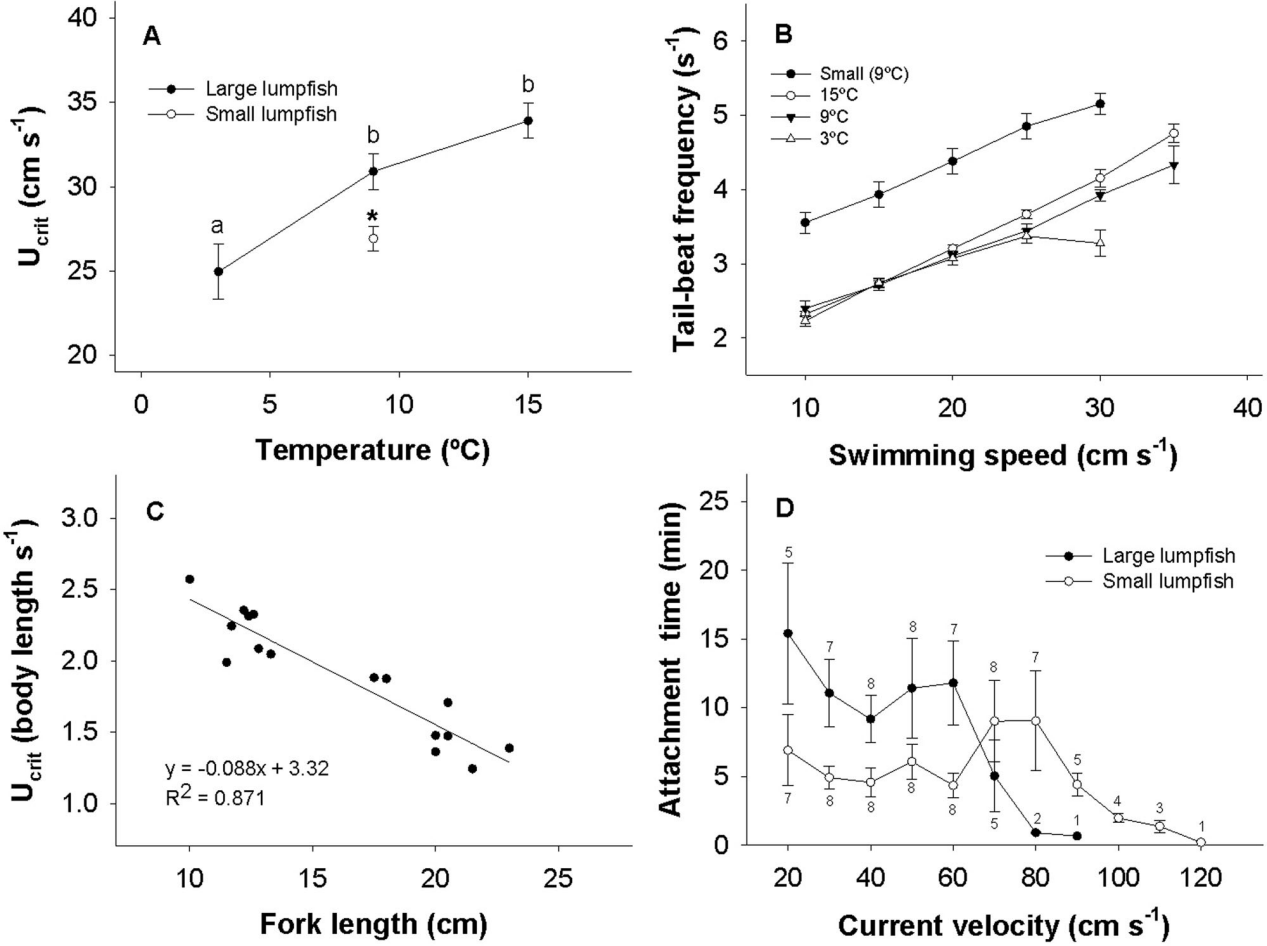
**Swimming performance of lumpfish.** (A) The absolute critical swimming speed (U_crit_) of lumpfish at different acclimation temperatures and size classes. (B) Tail-beat frequency of lumpfish as a function of swimming speed at different acclimation temperatures and size classes. (C) Scatterplot and linear regression of relative U_crit_ versus fork length of lumpfish at 9°C. (D) Longest observed attachment time as a function of current velocity in large and small lumpfish at 9°C. Relevant statistical differences are indicated with different symbols. *N*=8 in A, B and D. Numbers in D represent how many fish were observed to attach in specific current velocities. Data are mean±s.e.m.

The U_crit_ increased with temperature from 1.29±0.09 body lengths s^−1^ at 3°C to 1.55±0.09 and 1.67±0.06 body lengths s^−1^ at 9 and 15°C, respectively. In absolute terms, these U_crit_ values correspond to 25.0±1.6, 30.9±1.1 and 33.9±1.0 cm s^−1^ at 3, 9 and 15°C, respectively (Fig. 4A). The tail-beat frequency (TBF) increased steadily with swimming speed similarly at the three test temperatures, except at the highest swimming speeds, where fish at 3°C were unable to reach a similar maximum TBF as observed at the higher temperatures (Fig. 4B).

### Size effects and attachment behaviour

The weight, fork length (L_f_), condition factor, relative ventricular mass (RVM) and relative liver mass (RLM) of the experimental groups tested in the swim tunnel are summarized in Table 1. Larger lumpfish were ∼300 g and ∼20 cm, while smaller lumpfish were ∼75 g and ∼12 cm. Both RVM and RLM were statistically similar between groups with general values of 0.1% and 2.2% of whole body weight for RVM and RLM, respectively.

**Table 1.**
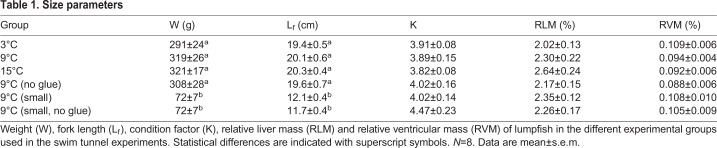
**Size parameters**

At 9°C the U_crit_ of smaller lumpfish was 2.24±0.07 body lengths s^−1^, corresponding to 26.9±0.7 cm s^−1^, meaning that smaller lumpfish had a slightly, but significantly lower absolute U_crit_, but a higher relative U_crit_ compared to the larger size class tested (Fig. 4A,C). The negative relation between relative U_crit_ and L_f_ are shown in Fig. 4C, where R^2^ of the linear regression was 0.871. When swimming at the same speeds, smaller lumpfish were required to use a higher TBF (Fig. 4B).

When giving lumpfish the opportunity to utilize their suction cup to avoid exhaustive swimming, a size dependent threshold in current tolerance was found, where the smaller lumpfish were able to remain attached in stronger water currents (Fig. 4D). Larger fish struggled to hang onto the swim chamber floor at 70 cm s^−1^, where two out of eight individuals managed to remain attached for barely 1 min at 80 cm s^−1^. At their maximum current velocity threshold, larger lumpfish were observed to slowly slide along the swim chamber floor on their suction cups until they hit the rear grid. The smaller lumpfish were not observed to slide on their suction cup, but instead released suddenly followed with brief burst swimming for a few seconds as a means of repositioning themselves. However, they would most often fail to reposition and instead get stuck on the rear grid of the swim tunnel. The current threshold of the smaller lumpfish varied from 80 to 110 cm s^−1^, where three individuals managed to remain attached for more than 1 min at 110 cm s^−1^.

At the initial current speeds, some individuals preferred to swim, while it was rare that fish remained attached for more than 20 min at any current speed. At moderate current velocities smaller lumpfish generally chose to utilize their suction cup for ∼5 min, while attachment times increased to ∼10 min in stronger currents prior to reaching their threshold for attachment. Larger lumpfish generally used their suction cup for up to 12 min before releasing themselves in moderate currents (Fig. 4D).

### Haematological parameters in response to exhaustive exercise stress

Cortisol levels were low and statistically similar between controls (5.5±0.7 ng ml^−1^) and immediately after the applied stressors, but increased significantly to 54.7±7.8 ng ml^−1^ after 1 h of recovery, and remained elevated at statistically similar values after 3 h. Lactate increased significantly from 0.06±0.01 mM in controls to 0.31±0.09 mM after the chase protocol, but returned to control values after 1 h of recovery. Plasma glucose, osmolality and Na^+^ were all statistically unaffected by sampling time. However, compared to the controls, plasma Cl^−^ decreased significantly by 6 mM following the stress trial, and decreased further by 13 mM in both recovery time points compared to controls. A slight progressive decrease in plasma K^+^ over time was found, with a significant difference of 1.2 mM between controls and the 3 h recovery group. Haematocrit (Hct) and haemoglobin concentration (Hb) did not change significantly between sampling times, where pooled averages were 17.4% and 0.65 mM for Hct and Hb, respectively. This resulted in a mean corpuscular haemoglobin concentration (MCHC) that was also unaffected by sampling time. All haematological measurements are summarized in Table 2.

**Table 2.**
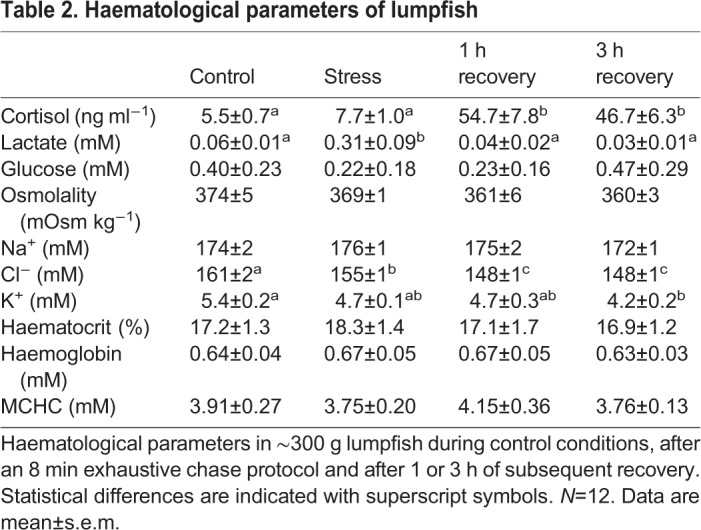
**Haematological parameters of lumpfish**

## DISCUSSION

### Aerobic capacity and thermal niche

In poikilothermic fish, SMR is expected to increase steadily with increasing acclimation temperature owing to acceleration of all biochemical processes and increased maintenance costs (Fry and Hart, 1948; Brett, 1964; Priede, 1985). In previously investigated fish species, the Q_10_ of the SMR has generally been found between 1 and 3 (reviewed by Lefevre, 2016). Hence, a Q_10_ of 1.7 reported here for the lumpfish from 3 to 15°C can be considered a normal thermal response.

The MMR, AS and U_crit_ increased at higher acclimation temperatures in lumpfish. Similar patterns have been found in several other species of fish investigated within ecologically relevant temperatures (e.g. Claireaux et al., 2006; Gräns et al., 2014; Norin et al., 2014; Hvas et al., 2017a). A reduced locomotory capacity in cold extremes of the thermal niche can be ascribed to a reduction in the maximum velocity of red muscle fibre shortening and lower power production (Rome, 1990; Rome et al., 1992). This is likely the case in lumpfish, since the maximum TBF was reduced at 3°C which resulted in a lower U_crit_ relative to the higher test temperatures.

A factorial AS of 2.3 to 2.8 within the tested thermal interval, as well as the measured SMR and MMR reported here, are in good agreement with two previous studies of lumpfish at 10-11°C at the same ontogenetic stage (Killen et al., 2007; Ern et al., 2016). Compared to more athletic species such as salmonids, lumpfish appear to have a low AS and a low factorial AS, mainly because of poor MMR. This can be illustrated by comparison to Atlantic salmon post smolts of ∼350 g at 13°C, where the AS was 406 mg O_2_ kg^−1^ h^−1^ and the factorial AS was 4.1 (Hvas et al., 2017b), while here the AS and factorial AS of ∼300 g lumpfish at 15°C was 173 mg O_2_ kg^−1^ h^−1^ and 2.3, respectively (Fig. 2A,B).

A low AS does not appear to be associated with smaller heart size since the RVM of lumpfish is similar to Atlantic salmon (Hvas and Oppedal, 2017). However, Hct and Hb concentration were generally low compared to most other fish (e.g. Caldwell and Hinshaw, 1994; Baker et al., 2005; Wells et al., 2005; Hvas et al., 2017b). This is suggestive of a low oxygen carrying capacity of the blood and reflects a low aerobic performance in lumpfish. Furthermore, the tail of lumpfish is small relative to the rest of the body, and lower muscle mass dedicated to swimming may also explain a lower AS and MMR compared to other species.

The upper limit of the thermal niche of lumpfish is 18°C, or perhaps slightly lower, since this temperature caused a high accumulated mortality over 3 weeks. In addition, cataract formation was observed at both 15 and 18°C, and has previously been reported in lumpfish at 16°C (Nytrø et al., 2014). Cataracts are well studied in farmed Atlantic salmon, and are similarly associated with chronic exposure in the upper limit of the thermal niche (Bjerkås and Sveier, 2004; Sambraus et al., 2017). The disruption of eyesight owing to heat-induced denaturation of proteins in the retina will obviously have profound negative consequences on foraging and predator avoidance.

However, cataracts in some individuals did not explain the high accumulated mortality at 18°C. Limitations in oxygen uptake and its delivery to respiring tissues are commonly discussed as causing the upper boundary of the thermal niche in fish (Brett, 1971; Farrell, 2002; Clark et al., 2013). At 18°C, the lumpfish were still eating while activity levels had increased notably to a hyperactive state compared to colder temperatures. Hence, it is unlikely that the lumpfish were limited in oxygen uptake and supply, since a compromised aerobic capacity should theoretically reduce appetite and activity levels, similar to hypoxic conditions (Claireaux et al., 2000; Remen et al., 2016a).

The underlying cause for the upper thermal niche boundary in the lumpfish is therefore uncertain, but is likely related to the behavioural changes in activity levels and swimming patterns observed in the holding tanks. These changes may be caused by alterations in nerve functioning, hormone balance and cognitive abilities induced by an extreme thermal environment, or may simply be a form of escape behaviour where lumpfish are searching for lower temperatures. Regardless of the physiological mechanisms involved, chronic exposure of 18°C is detrimental to the lumpfish owing to abnormal behaviour, cataract formation and high mortalities.

### Swimming capability and attachment behaviour

When considering the pelagic nature of lumpfish in parts of its life cycle (Blacker, 1983), the prolonged swimming capacity is strikingly poor compared to other pelagic fish of a similar size, where absolute U_crit_ generally is two to three times higher (e.g. Dickson et al., 2002; Claireaux et al., 2006; Hvas et al., 2017a). However, due to the globiform morphology of lumpfish in conjunction with poorly developed tail musculature, this is not a surprising result.

The intuitive size dependence of U_crit_, where smaller fish can swim more body lengths s^−1^, while larger fish have a higher U_crit_ expressed in absolute units, is similar to other species of fish (Brett, 1965; Remen et al., 2016b). Smaller lumpfish require a higher TBF to swim at similar speeds as larger individuals, and although they are able to attain higher TBF overall, they reach fatigue in lower current velocities. Consequently, smaller lumpfish will be less able to navigate in open waters with notable currents or turbulence.

When given the chance to utilize the ventral suction cup in the swim tunnel, some individuals preferred to swim at sub-U_crit_ speeds, while others would attach themselves to the bottom floor of the swim chamber for the majority of the time. At current velocities higher than their U_crit_, lumpfish were forced to utilize their suction cup to avoid getting stuck at the rear grid. Brief burst swimming was sometimes sufficient for repositioning, but generally when a lumpfish released itself it would end up getting stuck at the rear grid. In moderate current speeds of 30 to 60 cm s^−1^, it seemed random how long individual fish remained attached. This is exemplified by the large error bars in Fig. 4D, where attachment times could range from a few seconds to above 20 min, although 5-10 min was most common. These observations indicate that attachment times were voluntary and that subsequent release was a spontaneous decision. Hence, it did not occur to the lumpfish that current velocities had exceeded their swimming capacity and detachment could be dangerous either through collision damage or by being carried away to unfavourable habitats. In stronger current velocities, the maximum attachment time declined and became more consistent, which indicates that release eventually became non-voluntary. This shows that the ability of lumpfish to adhere onto surfaces is removed when currents become too strong. Furthermore, the hard and smooth plastic floor of the swim tunnel chamber should be an ideal substrate for attachment, meaning that attachment to other substrates encountered naturally in the marine environment may be less efficient (Imsland et al., 2015).

Interestingly, smaller lumpfish were able to remain attached in stronger currents. This suggests that higher drag forces experienced in larger fish owing to their larger cross-sectional areas was not properly compensated for by increases in suction force. Smaller lumpfish have poorer swimming capacities, and a stronger suction ability could therefore be important in earlier life stages when these fish are more vulnerable.

The unique ability to adhere onto surfaces could, in theory, be useful for both withstanding rough current conditions and to save energy. Lumpfish in the present study did not attempt to remain attached in moderate current velocities that exceeded their swimming capacities, but instead repeatedly detached spontaneously after brief periods of inconsistent durations. The main purpose of the suction behaviour is therefore more likely to be energy saving so that more resources can be diverted to growth. A similar conclusion was reached in a previous study on the foraging mode in lumpfish, where conditions with abundant food promoted suction behaviour, while conditions with sparse supply increased time spent actively foraging (Killen et al., 2007).

### Stress response

In the present study, lumpfish were subjected to an 8 min intensive chase protocol combined with air exposure, which should be more than sufficient to invoke all possible physiological responses associated with exercise stress. Curiously, the stress response, as assessed from haematological analyses, was modest and unlike many other species of fish investigated with similar approaches.

Baseline plasma cortisol was 5.5 and peaked at 54.7 ng ml^−1^ after 1 h of recovery. These values are several factors lower than reported in other fish such as striped bass and salmonids (e.g. Noga et al., 1994; Wagner et al., 2005; Kiessling et al., 2009; Hvas et al., 2017b), but similar to various species of sturgeon that have been described as having low endocrine capacities and modest responses to acute stressors (Maxime et al., 1995; Barton et al., 2000; Belanger et al., 2001).

In lumpfish, plasma glucose concentrations at rest were low and unaffected by the stress protocol. However, plasma glucose is generally expected to increase following stress, facilitated by the release of catecholamines into the blood, as an adaptive response to meet increased metabolic demands by mobilizing energy stores (Wendelaar Bonga, 1997).

In addition, lactate concentrations were barely detectable at rest, with a modest peak of 0.31 mM immediately following the stress test, and had returned to resting levels after just 1 h. In other species, plasma lactate concentrations may reach 5-15 mM following exercise stress, and subsequently take 6-12 h to return to control levels (Milligan and McDonald, 1988; Wood, 1991; Hvas et al., 2017b). However, it is possible that the majority of lactate was mobilized *in situ* in the white musculature, as is known to occur in some species (Milligan and McDonald, 1988; Wood, 1991). In any case, these reported values of plasma cortisol, glucose and lactate demonstrates the limited capability of lumpfish to respond to exhaustive exercise stress.

This apparent lack of a strong acute stress response is further supported by the apparent absence of EPOC following the U_crit_ trials, which suggests that swimming until maximum capacity causes negligible physiological disturbances to the lumpfish.

Likewise does the lack of change to Hct and Hb concentration, where stress may cause swelling of erythrocytes. Furthermore, other species recruit additional erythrocytes via splenic contraction as a response to exercise which causes notable increases to the Hct (Yamamoto, 1988; Kita and Itazawa, 1989).

Exhaustive exercise was expected to greatly increase plasma electrolyte levels owing to fluid shifts from extra- to intracellular compartments (Wood, 1991; Kieffer, 2000), and owing to increased passive ion uptake from the hyperosmotic seawater environment facilitated by increased branchial gas transfer. Evidently, this was not the case since plasma sodium concentration remained unaffected. Furthermore, contrary to expectation, plasma osmolality tended to decrease slightly, although not significantly, while plasma chloride progressively decreased significantly following exhaustive exercise and recovery. Plasma chloride decrease may be linked to active acid-base compensations, where chloride functions as a counter ion to bicarbonate in transepithelial exchange (Evans et al., 2005). However, although not assessed here, the metabolic acidosis encountered must have been minimal judging from the modest plasma lactate concentrations measured. Hence, the lumpfish is able to maintain osmotic integrity during exhaustive exercise stress. This is perhaps not a surprise when considering a presumably low anaerobic component and a low maximum rate of oxygen uptake, since these are the main drivers for osmotic disturbances during strenuous activity in other fish.

As hypothesized, the stress response of lumpfish was low. Many species of fish rely on high aerobic capacities and powerful burst swimming for migration, foraging and predator avoidance, and a strong stress response is therefore a crucial adaptation for survival. However, lumpfish have adapted to a more sedentary lifestyle where these traits are less important. Interestingly, the haematological effects and lack of EPOC in relation to stress presented here are generally similar to sturgeons (Barton et al., 2000; Kieffer et al., 2001; Baker et al., 2005), which are primitive fish that are sluggish benthic feeders very distantly related to lumpfish. A low aerobic capacity along with low anaerobic and endocrine responses to exercise stress may be a common adaption in fish with similar ecological traits.

## CONCLUSION

The lumpfish is a species that does not meet the expectation of a fish with a partly pelagic nature, as revealed here by a low AS, low U_crit_ and absence of a strong stress response to exhaustive exercise. Its unique ability to adhere onto surfaces appears to primarily be a tool to save energy rather than to withstand strong current conditions. Finally, lumpfish is a cold water species, and the upper thermal niche boundary of 18°C reported here is likely a key component in defining the geographical limit of its natural distribution where a normal life cycle can be completed.

## MATERIALS AND METHODS

### Lumpfish

Hatchery reared lumpfish were kept in large circular holding tanks at the Matre Research Station, Institute of Marine Research, Norway. Vertical oriented half-circular plastic pipes were provided in the tanks as additional substrates to accommodate attachment behaviour. Food pellets were provided daily to excess through automatic feeding devices. Stable temperatures and oxygen levels above 85% saturation were maintained in the holding tanks at all times through a constant high inflow from a large thermally regulated reservoir of filtered and UVC treated seawater of 34 ppt. A standard acclimation temperature of 9°C was used for the majority of the time, and was the only test temperature used for smaller lumpfish (∼75 g) and when assessing acute stress effects on haematological parameters. For larger lumpfish (∼300 g), additional holding tanks were maintained at either 3 or 15°C for a minimum of 3 weeks prior to swim tunnel trials at these respective temperatures. In lumpfish acclimated to 15°C, the temperature was further increased to 18°C for 3 weeks. However, due to high rates of mortality and abnormal behaviour at 18°C (Fig. 1), swim trials were not performed at this temperature. Instead, surviving fish were brought back to 9°C for an additional 3 weeks to further monitor behaviour and mortality. All experiments were performed between September 2017 and February 2018 in accordance with the Norwegian legislations on animal welfare in biological research under permit number 9776.

### Swim tunnel setup

A submerged 90 l intermittent-flow swim tunnel respirometer (Loligo Systems) was used to measure oxygen uptake rates (MO_2_) and U_crit_, and to assess attachment capabilities. The swim section of the setup was 20×19.5×66 cm. Water temperature was maintained by a constant open-flow through the buffer tank containing the respirometer, similar to the holding tanks. Water currents were generated by a motor driven propeller. A thorough description of the relationship between motor output and current velocity was obtained with a handheld flow meter (Hontzsch Flow Measuring Technology), so that desired current velocities could be generated in the swim trials. A fibre optic oxygen sensor and a temperature sensor were securely attached upstream of the swim section, while an inlet downstream of the propeller was connected to a powerful flush pump placed in the buffer tank that allowed for efficient and rapid flushing of the respirometer. These devices were connected to a computer program (AutoResp Respirometry Software; Loligo Systems) where measurements were logged every second during experimentation, while flushing automatically could be turned on and off in desired intervals. The oxygen sensor was calibrated every week according to the manufacturer's instructions. Black plastic sheets were used to cover up most of the setup to prevent visual disturbance of the fish. To further prevent unwanted disturbance during measurements, the entire setup was placed in a specifically allocated room where only the experimenter was present.

### Swim tunnel protocols

To minimize the confounding metabolic effects of specific dynamic action, food was withheld for 1 day before a fish was moved into the swim tunnel respirometer. Lumpfish show little or no behavioural response to the visual presence of humans, meaning that a fish could easily be netted from the holding tanks and transported to the swim tunnel setup within seconds. Once in the tunnel, MO_2_ measurements started. Here, the respirometer was kept closed off for 20 min and then flushed for 10 min to restore oxygen levels and remove waste products, which ensured that oxygen saturation did not fall below 95%. This cycle was repeated automatically for the entire duration of the experimental protocol. The fish was allowed to acclimate to the setup overnight, where propeller speed was kept at a bare minimum to ensure proper mixing of the water within the setup without enforcing swimming activity.

The following day, swim trials commenced. For the assessment of MMR and U_crit_ at different acclimation temperatures, the current velocity was first increased to 10 cm s^−1^, and then increased by an additional 5 cm s^−1^ every 30 min to allow for an MO_2_ measurement at varying swimming speeds. This stepwise increase was continued until the fish were unable to swim against the current. Current velocity was then quickly decreased to allow the fish to regain their swimming position and was subsequently increased again. Fatigue was defined as when the fish repeatedly fell back on the rear grid within seconds. The time was then noted, and current velocity was decreased again to a minimum. MO_2_ measurements were continued for an additional 2 h to assess EPOC. The fish were then removed from the setup and an MO_2_ measurement was made in the empty respirometer to account for possible bacterial respiration. After swim trials, fish were euthanized with a blow to the head, and weight, L_f_, width and height were recorded. In addition, the ventricle and liver were dissected out and weighed to account for RVM and RLM, respectively.

During steady swimming, the TBF was measured as the time to carry out 100 tail beats, and was recorded three times at each swimming speed. To enforce swimming and prevent attachment onto the swim tunnel surface, antibacterial surgical skin glue (Histoacryl; B. Braun Surgical) was applied on the ventral suction disc prior to transfer into the setup. This procedure was completed within seconds and anaesthesia was therefore deemed unnecessary. Eight replicate U_crit_ swim trials were completed at 3, 9 and 15°C for larger lumpfish.

When assessing current tolerance of the attachment behaviour, a similar protocol as described above was performed on fish without surgical glue on the suction disc. Here, current velocity was increased in steps of 10 cm s^−1^ either every 30 min if the fish remained attached for the entire interval, or when the fish had attached and released itself three times at a specific current speed. Timing began when the fish attached to the bottom of the swim chamber. Current speed was then increased accordingly until the lumpfish were unable to remain attached onto the bottom of the swim section for more than 1 min. At higher current velocities, detached lumpfish were often unable to reposition themselves, even with brief burst swimming. In these instances, current speed was then briefly decreased to allow for reattachment. Eight replicates were made at 9°C for larger lumpfish.

To assess size effects in U_crit_ and the capacity for attachment in strong currents, eight replicate swim trials (similar protocol as above) with lumpfish of a smaller size class were made at 9°C, both with and without surgical glue on the suction disc. However, since these fish were relatively small compared to the respirometer volume, a reliable trace in oxygen uptake could not be obtained within reasonable time, and MO_2_ measurements are therefore not reported for these groups.

### Calculation of metabolic rates and critical swimming speed

For each closed period, MO_2_ was calculated from the linear decrease in oxygen in the respirometry chamber as follows:

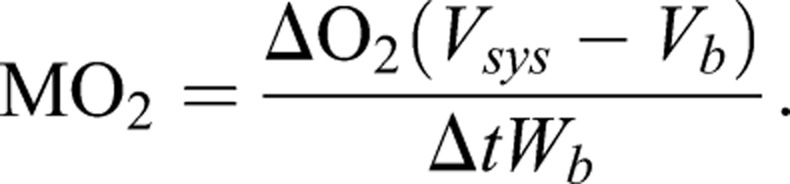


ΔO_2_ is the change in oxygen, Δt is the change in time, V_sys_ is the volume of the system, while V_b_ and W_b_ are the volume and weight of the fish where a density of 1 kg l^−1^ is assumed. While the fish acclimated to the setup overnight prior to the swim trial, several measurements of routine metabolic rates were obtained. An estimation of resting metabolic rate, termed SMR in fish and other poikilotherms, was made based on the average of the three lowest measurements during this period of approximately 18 h. MMR was defined as the highest measured MO_2_, which coincided with the highest swimming speed performed prior to reaching fatigue. AS was then calculated as MMR – SMR, while the factorial AS was calculated as MMR divided by SMR.

To express the rate change in MO_2_ between acclimation temperatures, the temperature quotient, Q_10_, was calculated as:

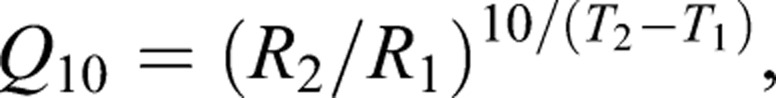


where R is MO_2_ and T is temperature.

U_crit_ was calculated according to Brett (1964):

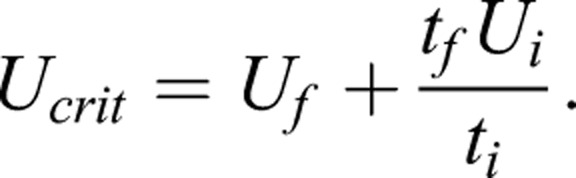


Here, U_f_ is the last completed current velocity, t_f_ is the time spent at the current velocity where fatigue was reached, t_i_ is the time spent at each velocity and U_i_ is the magnitude of the velocity increment.

Having an object within a water current of a fixed cross-sectional area obstructs the flow in such a way that the current velocity increases around the object. This is termed solid blocking, and the actual current velocity experienced by the fish in the swim tunnel can then be approximated according to Bell and Terhune (1970):

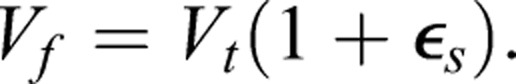


Here, V_f_ is the water velocity when a fish is present, V_t_ is the water velocity in the empty swim tunnel, and ɛ_s_ is the fractional error owing to solid blocking effects in V_t_.

ɛ_s_ is calculated as:

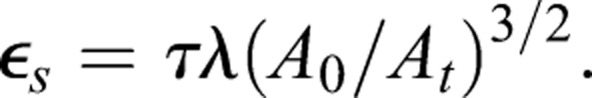


Here, τ is a dimensionless factor of 0.8, λ is a shape factor calculated as 0.7 (length/thickness) for ellipsoid shapes such as lumpfish, A_0_ is the cross-sectional area of the fish, and A_t_ is the cross-sectional area of the swim tunnel. A few of the larger lumpfish tested had cross-sectional areas relative to the swim tunnel exceeding 10%, which imposes an appreciable theoretical current increase. For a consistent comparison, all U_crit_ values reported have therefore been compensated for solid blocking effects.

### Stress test and haematological analyses

To assess the effects of exhaustive exercise stress on haematological parameters, individual lumpfish were netted from the holding tank and moved into a small 100 l tank. Here, they were manually stressed for 8 min by a combination of chasing within the tank and air exposure to ensure physiological exhaustion. A 1.5 ml blood sample was then drawn from the caudal vein with a heparinized syringe either immediately after the stress test, or following 1 or 3 h of recovery in a holding tank, after having been knocked unconscious with a blow to the head. Twelve replicates were made for each time period, and in addition, 12 blood samples were drawn from lumpfish netted directly from the holding tank to serve as controls.

After blood sampling, the Hct was measured in duplicates as the fraction of red blood cells from small subsamples centrifuged in capillary tubes (StatSpin MP Centrifuge), and the Hb was measured from a 10 µl subsample with a Hb assay kit (MAK115; Sigma-Aldrich). The remaining blood was then centrifuged at 5000 ***g*** for 5 min to obtain blood plasma, which was stored at −80°C for later analyses.

Concentrations of plasma Na^+^, K^+^ and Cl^−^ were measured with an electrolyte analyser (Cobas 9180; Roche Diagnostics). Plasma osmolality was measured with a Fiske 210 Micro-Sample Osmometer (Advanced Instruments). Plasma lactate and glucose concentrations were measured spectrophotometrically with MaxMat PL (MaxMat). Cortisol was measured with an ELISA kit (IBL International GmbH) and a Sunrise microplate reader (Tecan).

### Statistics

To test for effects of acclimation temperature, as well as for sampling time in the stress test, a one-way ANOVA was used followed by a Tukey's *post hoc* test to determine which groups differed. Equal variance was confirmed with Levene's test. For assessment of when MO_2_ returned to pre-swim values following the U_crit_ protocol, a repeated measures-ANOVA with Dunnett's test was used. When comparing swimming performance between the two size-classes, a Student's *t*-test was used (Sigmaplot 12.3; Systat software). A *P*-value below 0.05 was considered significant. Data are presented as mean±standard error of the mean, unless otherwise specified.
